# Spatially Non-Stationary Response of Carbon Emissions to Urbanization in Han River Ecological Economic Belt, China

**DOI:** 10.3390/ijerph20010363

**Published:** 2022-12-26

**Authors:** Weisong Li, Zhenwei Wang, Zhibin Mao, Jiaxing Cui

**Affiliations:** 1 Collaborative Innovation Center for Emissions Trading System Co-Constructed by the Province and Ministry, Wuhan 430205, China; 2 Hubei University of Economics, Wuhan 430205, China; 3 College of Public Administration, Hubei University, Wuhan 430062, China; 4 Experimental Teaching Centre, Hubei University of Economics, Wuhan 430205, China; 5 College of Urban and Environmental Sciences, Central China Normal University, Wuhan 430079, China

**Keywords:** carbon emissions, urbanization level, geographically weighted regression model, multiscale analysis, China

## Abstract

Within the context of the “30·60 dual carbon” goal, China’s low-carbon sustainable development is affected by a series of environmental problems caused by rapid urbanization. Revealing the impacts of urbanization on carbon emissions (CEs) is conducive to low-carbon city construction and green transformation, attracting the attention of scholars worldwide. The research is rich concerning the impacts of urbanization on CEs but lacking in studies on their spatial dependence and heterogeneity at multiple different scales, especially in areas with important ecological statuses, such as the Han River Ecological Economic Belt (HREEB) in China. To address these gaps, this study first constructed an urbanization level (UL) measurement method. Then, using a bivariate spatial autocorrelation analysis and geographically weighted regression model, the spatial relationships between UL and CEs from 2000 to 2020 were investigated from a multiscale perspective. The results were shown as follows. The total CEs in the HREEB witnessed an upsurge in the past two decades, which was mainly dispersed in the central urban areas of the HREEB. The ULs in different regions of the HREEB varied evidently, with high levels in the east and low levels in the central and western regions, while the overall UL in 2020 was higher than that in 2000, regardless of the research scale. During the study period, there was a significant, positive spatial autocorrelation between UL and CEs, and similar spatial distribution characteristics of the bivariate spatial autocorrelation between CEs and UL at different times, and different scales were observed. UL impacted CEs positively, but the impacts varied at different grid scales during the study period. The regression coefficients in 2020 were higher than those in 2000, but the spatial distribution was more scattered, and more detailed information was provided at the 5 km grid scale than at the 10 km grid scale. The findings of this research can advance policy enlightenment for low-carbon city construction and green transformation in HREEB and provide a reference for CE reduction in other similar regions of the world.

## 1. Introduction

Since the Industrial Revolution, human activities have generated numerous greenhouse gas emissions, which are dominated by carbon dioxide [1]. Overexploitation has led to society approaching or already exceeding “planetary boundaries” in some dimensions [2]. Extreme climate events, such as droughts, floods, and rising sea levels, are frequently reported [3]. Within this context, the United Nations adopted the 2015–2030 Sustainable Development Goals (SDGs) in 2015 [4], striving to provide sustainable benefits for present and future generations [5]. Subsequently, the Paris Agreement set a goal of keeping the global temperature rise well below 2 °C above pre-industrial levels this century, which further emphasized the importance of mitigating climate change [6,7,8]. In 2016, Habitat III launched the New Urban Agenda, aiming to measure how sustainable urbanization underpinned the SDGs [9]. Xi Jinping, president of the People’s Republic of China, proposed that “China will strive to contribute autonomously to peak carbon dioxide emissions before 2030 and achieve carbon neutrality before 2060” at the United Nations General Assembly in 2020. The proposal of the “30·60 dual carbon” goal has pushed low-carbon development and green transformation to the crossroads of a historical turning point [10].

Globally, the past decades have witnessed rapid growth, and urbanization, a typical symbol of modernization, has played an indispensable role in economic growth [6]. In 2007, the global urbanization rate exceeded 50% for the first time, indicating that the urban population exceeded the rural population [6]. As the world’s most populous country, China realized an urbanization rate exceeding 50% in 2011 [11]. Over the past 70 years, China has achieved the feat of moving half of its population to cities and towns, and since the 21st century, China’s urbanization rate has increased by approximately 30%, from 36.5% to 64.7%, and is predicted to reach 81.6% within the next three decades [11,12]. Meanwhile, urbanization is also viewed as the key driver affecting carbon emissions (CEs) [13,14,15,16]. Currently, the top 600 cities worldwide are inhabited by 20% of the global population, create about 60% of the GDP, and emit about 70% of global greenhouse gases [9]. Studies have demonstrated that rapid urbanization can lead to the deterioration of habitat conditions and the decline of ecosystem structure and function [17]. Undoubtedly, urbanization has exacerbated the dilemma of economic development and environmental protection [18,19,20]. Accelerated urbanization in China, especially in the form of the emergence of urban sprawl, has become a common problem among Chinese cities; it marks socio-economic development but also brings about ecological problems that cannot be neglected, especially excessive CE issues [21,22].

Urbanization is a prevalent phenomenon in the progress of modern civilization [23], which attracts the attention of scholars [24,25]. Currently, studies on urbanization mainly focus on the appearance of urban sprawl [26], urban development [27], urbanization level (UL) measurement [5], and the impact of urbanization on the eco-environment [1,28,29]. To quantify urban sprawl, indices such as average population density, single-, and multi-dimension urban sprawl have been proposed [26,30,31,32]. Specifically, Gielen et al. (2018) obtained a single urban sprawl index with a Bayesian factor analysis [30] that advanced traditional approaches to obtain the uncertainty of the inferred index. Du et al. (2021) constructed a single index to reflect the extent of urban sprawl by combining population data with built-up areas or average population density [26]. The research on the impact of urbanization on the eco-environment is mainly concentrated on their interaction [28], mechanism [29], and coupling and coordination analyses [29]. Meanwhile, research on UL and CEs has gained attention over the years [33,34]. The measurement of UL mainly includes population, economic, spatial, and social urbanization [35,36], among which population urbanization is the most preferred. Urbanization is one of the main factors contributing to global greenhouse gas emissions [37,38]. However, few studies on the impact of urbanization measured in an integrated manner with CEs have been conducted [39]. This study addresses the gap by comprehensively considering the population, economic, and land urbanization subsystems to construct a UL measurement method.

There are different views about the impact of urbanization on CEs [40,41,42]. One view is that urbanization accelerates the growth of CEs mainly by affecting residential and industrial energy consumption, construction industry energy consumption, and forest conversion for urban development [43,44]. Research by Liu and Bae confirmed that a 1% augmentation of China’s urbanization increased its CEs by 1.0% [45]. A study on China also supported the view that land urbanization and economic urbanization positively stimulated CEs by encouraging energy consumption [46]. Another view was that urbanization played a positive role in meeting CE reduction targets [47]. The idea that urbanization could effectively promote energy efficiency improvement was put forward [48]. Wang et al., analyzed the impacts of urbanization on CEs in OECD high-income countries and concluded that, with each percentage point increase in the urbanization rate, CEs per capita decreased by 0.015% [6]. Furthermore, some scholars found that there was an inverted, *U*-shaped relationship between urbanization and CE efficiency. In the early stage, urbanization promotes CE reduction, and as the process progresses, the positive impact becomes negative [49]. In general, there is a correlation between UL and CEs, but the relationship varies from different perspectives and at different stages of development. Therefore, it is necessary to reveal the spatial non-stationary characteristics of the impact of multi-scale urbanization on CEs, which can not only further understand the multi-scale characteristics of the impact of urbanization on CEs, but also provide decision-making support for low-carbon urbanization.

In this study, the Han River Ecological Economic Belt (HREEB) was selected as a case study. With a favorable geographical location in China, the HREEB is an important ecological barrier for China and has witnessed rapid urbanization in recent years [50]. Located in the transitional zone of the north and south floras and at the intersection of the east and west floras, the HREEB plays an important ecological role. As the water source of the middle route of the South-to-North Water Diversion project, the HREEB is also an important green ecological barrier in China and an important force for protecting China’s water safety. The purpose of the ecological economic belt is to explore a pilot zone where economic development and ecological protection can coordinate. Thus, it is important to examine the impacts of urbanization on CEs in the HREEB from a multiscale perspective, an aspect that previous research has rarely touched on, which can provide a theoretical reference for urban planning and a green economy for sustainable development.

To explore the impact of urbanization on CEs, this study evaluates the UL and CEs at the 5 km and 10 km grid scales, respectively. Then, using a bivariate spatial autocorrelation analysis and geographically weighted regression model, the spatial relationships between UL and CEs from 2000 to 2020 are explored from a multiscale perspective. The following questions are raised in this study: (1) What are the spatiotemporal patterns of CEs in the HREEB at different scales? (2) What are the spatiotemporal patterns of UL in the HREEB at different scales? (3) What are the spatial dependence and spatial heterogeneity between UL and CEs at different scales? The solution to the above problems may be conducive to harmonizing urbanization and low-carbon transformation in the HREEB and may simultaneously pave the way for low-carbon sustainable development from a global perspective of carbon neutrality in similar regions of the world. The content of this study is arranged as follows. The first section is an introduction to the research progress of urbanization and CEs. The second section presents the materials and methods used. The third section displays the results. The fourth part is a discussion of the study. The last section is a summary of the study.

## 2. Materials and Methods

### 2.1. Study Area

**Figure 1:**
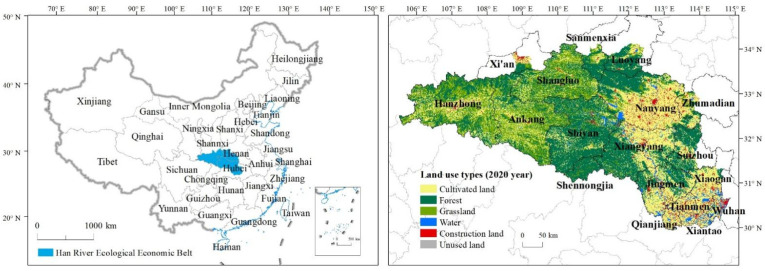
Study area in China.

The Han River Basin is rich in natural resources and has a solid economic foundation, profound cultural heritage, superior ecological conditions, and geographical advantages with outstanding ecological barrier function. The HREEB is a strategic route connecting the Yangtze River Economic Belt and the New Silk Road Economic Belt (Figure 1). It is also an important channel connecting the Yangtze River Economic Belt and the Silk Road Economic Belt in northwest China because of its unique geographical advantages. It is situated at the junction of the central and western regions of China. In addition, the HREEB is a major grain-producing area in China and an important base for the automobile industry, equipment manufacturing, and textile and garment production in the country, showing a good development trend. The middle and upper reaches of HREEB belong to the biodiversity ecological function zone of Qinling and Bao-Pakistan and the water source area of the Middle Route of the South-to-North Water Diversion project. As an important ecological barrier in the central and western regions, the UL of the lower reaches is relatively high. Coordinating urbanization and CEs has become a difficult problem for the sustainable development of the HREEB. To realize the green and sustainable development of the HREEB, it is necessary to reveal the multi-scale spatial non-stationary characteristics of UL and CEs in the HREEB.

### 2.2. Data Sources and Processing

The CE dataset in this study was obtained from the Open-Data Inventory for Anthropogenic Carbon Dioxide released by global grid data (https://db.cger.nies.go.jp/dataset/ODIAC/, accessed on 1 January 2022) [51]. This is a high-spatial-resolution global emission data product on CEs mainly based on fossil fuel combustion. Since the platform mainly publishes monthly data, the annual data we counted was mainly obtained by summing the data of each month. The calculation of UL involved population density, economic density, and the proportion of construction land. Land use data at a 30 km resolution, a 250 m resolution DEM, and a 1 km resolution population density and economic density data from 2000 and 2020 were obtained from the Data Center for Resources and Environmental Sciences, Chinese Academy of Sciences (http://www.resdc.cn, accessed on 1 January 2022). Due to a lack of population density and economic density data for 2020, we used the data for 2019 instead. To deeply reveal the impacts of urbanization on CEs, this study conducted research on the impact of UL on CEs from 5 km and 10 km scales, breaking from the traditional administrative scale [52,53,54,55].

### 2.3. Urbanization Level Measurement

According to the definition of urbanization, previous studies on the measurement of UL have usually portrayed four aspects: population, economic, spatial, and social urbanization [56,57]. Among them, population urbanization is a landmark characteristic of the urbanization process and is usually characterized by population density. Economic density, as the driver of urbanization, is usually characterized by economic urbanization. Spatial urbanization can provide a spatial guarantee for urbanization and is usually characterized by the proportion of construction land area. Social urbanization, as the final result of urbanization, is relatively macroscopic and difficult to quantify, especially at the grid scale. Therefore, social urbanization was not considered in this study. Meanwhile, since the first three are equally important, this study standardized these three indicators using standard deviation standardization, and assigned them the same weight to calculate the UL [25,58]. The calculation equation was as follows:

(1) $$UL_{i}=(ED_{i}+PD_{i}+DLP_{i})/3$$

where *UL_i_* denotes the UL of grid cell *i*; and *ED_i_*, *PD_i_*, and *DLP_i_* represent the economic density, population density, and construction land proportion of grid cell *i*, respectively.

### 2.4. Spatial Autocorrelation Analysis

A spatial autocorrelation analysis includes the two aspects of a global autocorrelation analysis and a local autocorrelation analysis and is often applied to detect the potential interdependence between geographic data within a region [54,55,59]. The core of the exploratory spatial data analysis approach is to explore whether an attribute value exhibits the characteristics of distribution clustering and spatial anomaly in space [60]. Spatial association patterns (convergence or heterogeneity) are generally measured and examined with the global Moran’s *I* index and the local Moran’s *I* index (LISA), which reveal the spatial distribution characteristics of objects [61]. In a global autocorrelation analysis, the most commonly used statistic is global Moran’s *I* (global Moran’s index), which is mainly used to describe the average degree of correlation between all spatial units and surrounding areas in a whole region. The equation is as follows:

(2) $$Moran^{\prime }s\;\;\;I=\frac{n\sum_{i=1}^{n}\sum_{j\neq 1}^{n}W_{ij}\cdot \left(X_{i}-\bar{X}\right)\cdot \left(X_{j}-\bar{X}\right)}{\left(\sum_{i=1}^{n}\sum_{j=1}^{n}W_{ij}\right)\sum_{i=1}^{n}{\left(X_{i}-\bar{X}\right)}^{2}}$$

where *X_i_* and *X_j_* denote the observed values in sampling plots *i* and *j*, respectively, $\bar{X}$ is the average value of *X*, and *W_ij_* is a spatial weight matrix (*ij* = 1, 2, 3, …, *n*). *Moran’s I* generally takes a value between −1 and 1. When *Moran’s* *I* < 0, it means that the spatial entities are discretely distributed, and a negative autocorrelation exists; when *Moran’s I* > 0, it indicates a positive autocorrelation. If *Moran’s I* approaches 0, it indicates that the space follows a random distribution, and a *p*-value is commonly used for a significance test.

Referring to Anselin and Rey, this study proposed a regional spatial autocorrelation analysis to explore the spatial relationship between UL and CEs [61]. The equation is as follows:

(3) $$I_{kl}^{i}=z_{k}^{i}\sum_{j=1}^{n}W_{ij}Z_{l}^{j}$$

where *W_ij_* is the spatial connection matrix between the spatial units *i* and *j*; $z_{k}^{i}=\frac{X_{k}^{i}-\bar{X_{k}}}{e_{k}}$; $z_{l}^{j}=\frac{X_{l}^{i}-\bar{\;X_{l}}}{e_{l}}$; $X_{k}^{i}$ is the value of attribute *k* of spatial unit *I*; and $X_{l}^{i}$ is the value of attribute *l* of spatial unit *j*. Moreover, $\bar{X_{k}}$ and $\bar{X_{l}}$ are the average values of attributes *k* and *l*, respectively; *e_k_* and *e_l_* are the variances of attributes *k* and *l*, respectively. Based on the local Moran’s *I* index, the calculation results were divided into four categories: H-H (high CEs and high UL-type area), L-L (low CEs and low UL-type area), H-L (high CEs and low UL-type area), and L-H (low CEs and high UL-type area).

### 2.5. Geographically Weighted Regression Model

A GWR model, an extension of the traditional regression analysis method [52,62], is an effective way to explore the impacts of urbanization on CEs because it considers spatial heterogeneity and it almost eliminates spatial autocorrelation [63,64]. Specifically, the equation is as follows:

(4) $$y_{i}=\beta_{0}\left(u_{i},v_{i}\right)+\sum_{k=1}^{p}\beta_{k}\left(u_{i},v_{i}\right)x_{ik}+\varepsilon_{i}$$

where *y_i_* is the dependent variable, *x_ik_* is the influencing factor, and *ε_i_* is the random error of sample *i*. Moreover, *β_j_*(*mi*, *ni*) is the regression coefficient *j* of sample *i*, the positive and negative of which represent the promoting or inhibiting effect of *x_ij_* on *y_i_*, respectively. In this study, *y_i_* refers to CEs, and *x_ik_* includes elevation, land use intensity, and UL. Since we focused on the effect of UL on CEs, the other two factors were, thus, listed as control variables.

## 3. Results

### 3.1. Spatiotemporal Patterns of Carbon Emissions

**Figure 2:**
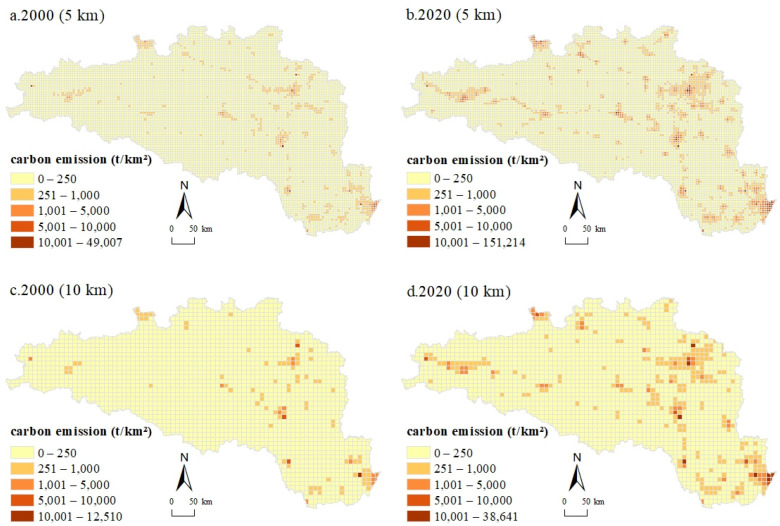
Spatiotemporal patterns of CEs at 5 km and 10 km grid scales in 2000 and 2020 in the HREEB.

Figure 2 shows the spatiotemporal distribution of CEs at 5 km and 10 km grid scales in 2000 and 2020. To effectively avoid the differences caused by the size of the scale unit, this study used the total CEs of the zonal statistics divided by the area of the grid to characterize the CEs. The total CEs in the HREEB witnessed an upsurge in the past two decades, with 1.664 × 10^7^ t in 2000 and rocketing to 4.587 × 10^7^ t in 2020, almost tripling in value. Generally, CEs were mainly dispersed in the central urban areas of cities within the HREEB. Wuhan and Xiaogan in the east, Jingmen in the south, Xiangyang and Shiyan in the middle, Hanzhong in the west, and Nanyang in the north were the concentration areas of CEs. At the 5 km grid scale for 2000, the distribution of CEs was more scattered and sporadically distributed throughout the region, with the east, central, and northeast areas being more concentrated, the west being more scattered, and the southwest being the least distributed (Figure 2a). At the 10 km grid scale, the CE distribution was relatively concentrated but still showed a certain dispersion characteristic (Figure 2c). Compared with 2000, at the 5 km grid scale, the CE distribution of the HREEB in 2020 was still scattered but showed an obvious expansion trend (Figure 2b). The central and northeast parts were still the most concentrated areas in terms of CEs, while obvious expansion was observed in the central and western regions. At the 10 km grid scale, CEs showed an obvious dispersed distribution trend compared with that in 2000, and the grid of high CE values increased significantly, while the southwestern part with low CEs also started to scatter high CE grids (Figure 2d).

### 3.2. Spatiotemporal Patterns of Urbanization Level

**Figure 3:**
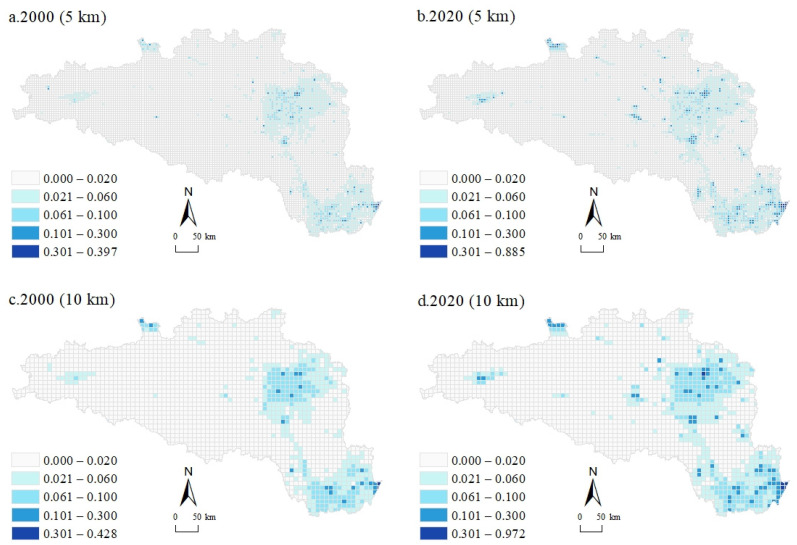
Spatiotemporal patterns of UL at 5 km and 10 km grid scales for 2000 and 2020 in the HREEB.

Figure 3 shows the spatiotemporal patterns of UL at 5 km and 10 km grid scales in 2000 and 2020. In 2000, the UL of the HREEB was 0.0122, while in 2020 was 0.0168, showing significant growth. To more intuitively represent the spatial characteristics of UL distribution, this study divided UL into five intervals. Generally, the ULs in different regions of the HREEB varied significantly, with high levels in the east and low levels in the central and western regions, while the overall UL in 2020 was higher than that in 2000. In 2000, the spatial distribution of UL differed significantly, with the higher-level grid mainly concentrated in Wuhan, Qianjiang, and Xiantao in the southeast, Nanyang and Xiangyang in the northeast, and generally lower ULs in the west. Specifically, at the 5 km grid scale for 2000, the patterns of UL were scattered, with the southeast and the northeast clustering a large number of rasters with high ULs (Figure 3a). In the western region, high UL grids were rare, and there were basically no areas with high ULs in the Shennongjia region in the southwest. At the 10 km grid scale, the patterns of UL were still scattered, but a certain distribution pattern was demonstrated (Figure 3c). In regions with high ULs, the surrounding ULs appeared to decrease regularly with distance. This could also be observed at the 10 km grid scale in 2020 (Figure 3d). In comparison, the number of regions with high ULs increased significantly in 2020 and continued to expand around the original regions with high ULs. Either at the 5 km grid scale or at the 10 km grid scale, grids with high ULs were more scattered in 2020 than in 2000. Over the past 20 years, the HREEB has experienced rapid economic development and a corresponding increase in UL, owing much to its excellent district and geographical conditions. The spatiotemporal patterns of UL have become more fragmented, as many sparsely populated areas have been exploited for development purposes.

### 3.3. Bivariate Spatial Autocorrelation Analysis

At the 5 km grid scale, the global bivariate Moran’s *I* values of UL and CEs in 2000 and 2020 were 0.174 and 0.232, respectively, while at the 10 km grid scale, the global bivariate Moran’s *I* values of UL and CEs in 2000 and 2020 were 0.264 and 0.336, respectively. Both were significant at the level of 0.0001. During the study period, there was a significant, positive spatial correlation between UL and CEs, indicating that the improvement in UL increased CEs. It could be seen that, at the 10 km grid scale, the global bivariate Moran’s *I* value was higher than that at the 5 km grid scale for both years. However, at both grid scales, the global bivariate Moran’s *I* values in 2020 showed significant increases over 2000, indicating that the spatial correlation changed more significantly over time. The bivariate spatial autocorrelation test indicated a significant spatial dependence effect between CEs and UL, generating a significant, positive externality during the study period. 

**Figure 4:**
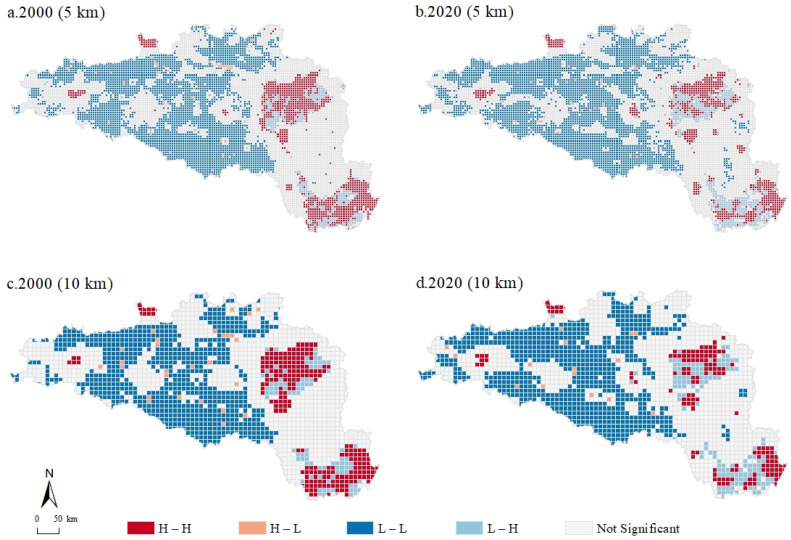
LISA maps of urbanization elements and ESBI at the 5 km grid scale in the HREEB in China.

Figure 4 shows the bivariate local spatial autocorrelation LISA clustering maps of CEs and UL at 5 km and 10 km scales between 2000 and 2020 in the HREEB. By comparing these maps, some similar spatial characteristics could be observed. Generally, the HREEB was dominated by L-L and H-H areas, and the spatial distribution showed an obvious two-level differentiation. H-H areas were mainly distributed around large cities in the southeast and northeast regions of the HREEB with wide plains, concentrated populations, and high ULs. L-H areas were surrounded, and there were essentially no H-L areas. In the central and western regions where forest resources are rich and the population is sparse, L-L areas were widespread, with a small number of H-H and H-L areas sporadically distributed, and there were essentially no L-H areas. At the 5 km grid scale, the distribution of each type of area was fragmented and staggered, whereas at the 10 km grid scale, the spatial distribution pattern was more obvious, which also verified that the global bivariate Moran’s *I* value was higher in 2020 than in 2000. Compared with 2000, in 2020 there was a certain decrease in H-H areas and an increase in L-H areas in regions where the original H-H areas were concentrated, and there were basically no H-L areas. The western region was still dominated by L-L areas, but their number decreased; H-L areas also showed a decrease, while H-H areas increased slightly. 

### 3.4. Impact of Urbanization Level on Carbon Emissions

**Figure 5:**
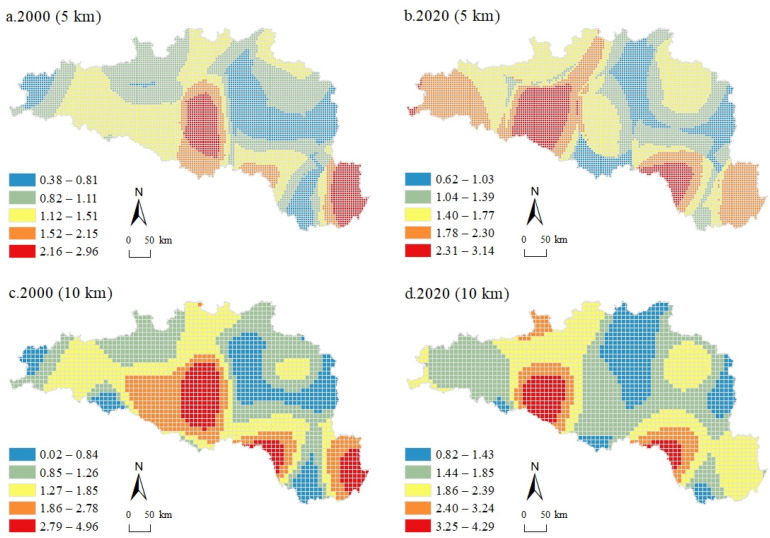
Spatial distribution of the regression coefficients of the effect of UL on CEs at 5 km and 10 km scales in the HREEB in 2000 and 2020.

We used OLS models to test for multiple covariance in the influencing factors of CEs (e.g., elevation, land use intensity, and UL) and found that the variance inflation factors were all less than 2. Moreover, the R^2^ value obtained by the GWR model was greater than that obtained by the OLS model. The Moran’s *I* value of residual CEs was significantly lower than the Moran’s *I* value of CEs, which indicates that the GWR model could solve spatial dependence to a large extent. Figure 5 shows the spatial distribution of the regression coefficients of the effect of UL on CEs at 5 km and 10 km grid scales in the HREEB in 2000 and 2020 using the GWR model. During the study period, UL had a positive correlation with CEs, but the impacts of UL on CEs varied at different grid scales. Numerically, the regression coefficient was higher in 2020 than that in 2000 for both grid scales, while it was lower at the 10 km grid scale than at the 5 km grid scale in the same year. Spatially, the distribution of regression coefficients showed a decreasing pattern from high values to the outer circle that was more obvious at the 10 km grid scale than at the 5 km grid scale. Moreover, the spatial distribution of regression coefficients was more scattered in the 5 km grid scale than in the 10 km grid scale, indicating that the impact of UL on CEs at a large scale was more stable in the HREEB. The regression coefficients of Shennongjia in the southwest region were larger, regardless of the scale and year. It may be because the region had a low UL and a better ecological environment, coupled with government policies to protect regions with fewer human activities; thus, a slight increase in UL could increase CEs. Low regression coefficients were sporadically distributed in the HREEB, while low regression coefficients were consistently maintained in the northwestern region. At the 5 km grid scale, the area with high regression coefficients tended to move westward, and the range was expanded. In 2000, the regression coefficients of the western region were essentially low, while by 2020, high regression coefficients accounted for a large proportion. A similar change pattern could be observed at the 10 km grid scale with a more regular pattern. Overall, the spatial fluctuation in the regression coefficients was large, which indicates that the impact of UL on CEs in the HREEB was unstable. This also corresponded to the differences in UL in different regions of the HREEB.

## 4. Discussion

### 4.1. Spatial Relationship between Urbanization Level and Carbon Emissions

The results showed that UL was positively correlated with CEs at 5 km and 10 km scales in the HREEB in 2000 and 2020, indicating that improvements in UL could increase CEs. This was mainly because the agglomeration of population and economy in the process of urbanization inevitably leads to the expansion of construction land and energy consumption, which inevitably causes increases in CEs [39,40]. Meanwhile, with the continuous progress of technology, the improvement of energy utilization efficiency reduces CEs to a certain extent [49]. Moreover, the HREEB was dominated by L-L and H-H areas, which also indicates that UL could accelerate the growth of CEs in the HREEB. We hold the same view that the regional differences in China’s urbanization process are not only on a large scale in the east, middle, and west, but also within these regions [65,66]. The ULs in different regions of the HREEB varied significantly, with high levels in the east and low levels in the central and western regions. The main reason could be that the southeast and northeast are located in the Jianghan Plain area, where the topography is flat and the climate is pleasant, making it suitable for the development of agriculture and various industrial economies, as well as gathering a large population [50]. In contrast, the western region is located in the Qinba Mountains, where the terrain is mainly mountainous with extensive vegetation and a sparse population, the development of urbanization is slow, and the level of urbanization is relatively low. The spatial distribution of CEs was similar to that of urbanization in the HREEB. Generally, CEs were mainly dispersed in the central urban areas of cities within the HREEB. It was concluded that CEs were mainly located in areas where ULs were high. 

Studies on the relationship between urbanization and CEs have been favored by the academic community, and a large amount of research has been conducted with varying conclusions [15,67]. Previous studies have found that there are negative, positive, or “inverted *U*-shaped” relationships between urbanization and CE intensity [39,40,41,42]. The HREEB is an important ecological barrier in China, and although urbanization is being promoted rapidly, there is still a large gap in its urbanization development level compared with economically developed regions. In addition, while technology can increase the efficiency of energy consumption and, thus, reduce CEs to some extent [50], it is not enough to offset the incremental increase caused by rapid urbanization. Therefore, a positive correlation was still present overall in the HREEB. Additionally, there were certain differences in the distribution rules under different research scales, which prompted us to consider the following question: Is there any difference in the impacts of UL on CEs under different research scales? According to the results of the GWR model, the spatial distribution of the regression coefficients of the impact of UL on CEs exhibited a certain pattern that was more obvious at the 10 km grid scale than at the 5 km grid scale. This finding is consistent with the pattern where variability is more pronounced at small scales and spatial distribution patterns are more significant at large scales [39]. Thus, for control of the overall law, a large scale was more appropriate, and small-scale research was needed to refine the differences between regions. In addition, the distribution difference laws at different scales could provide scientific support for differentiated policy formulation and management implementation.

### 4.2. Policy Implications

Urbanization is the inevitable result of modern civilization and economic development, and the promotion of urbanization inevitably brings population growth and the intensification of energy consumption [15,16]. Energy consumption is the most direct and most important cause of CEs. Admittedly, it is unrealistic to curb the urbanization process to reduce CEs and, thus, achieve the “30·60 dual carbon” goal, which is also out of phase with the law of social development. Our results showed that the significant, positive relationship between UL and CEs in the HREEB implied that the impact of accelerated urbanization on CEs was self-evident. Based on the results of this study, policy implications are presented for the following aspects.

(1) For the urbanization process, we know that the measurement of UL includes the three aspects of population, economy, and construction land [35], which indicates that population transfer to urban areas, accelerated economic development, and the continuous expansion of construction land are all important drivers for the increase in CEs. Thus, it is possible to assess these aspects to make relevant policy restrictions. For example, from the perspective of policy makers, controlling the size of the urban population and promoting the low-carbon utilization of land can improve efficiency [68] by regarding the urbanization process as an opportunity for low-carbon development to control the growth rate of CEs while ensuring economic development.
(2) To improve energy efficiency and reduce CEs, considering that urbanization inevitably leads to an increase in CEs, reducing CEs by improving technology and adjusting the energy structure is the most feasible way [69,70]. Studies have proved that the technological level of a country is linked to energy efficiency, and improvements in the technological level can reduce carbon dioxide emissions by improving energy efficiency [71,72]. In addition, energy restructuring through the use of wind, solar, and other clean energy sources to substitute for high-CE fuels, such as coal, to achieve CE reductions at the source have been advocated [73,74]. This is, in fact, a method of advancing technology. Thus, the central urban areas of cities in the HREEB, such as Wuhan and Xiaogan in the east, Jingmen in the south, Xiangyang and Shiyan in the middle, Hanzhong in the west, and Nanyang in the north, were concentration areas of CEs, where CEs were mainly dispersed and should be improved at the technological level.
(3) Energy consumption reductions should meet emission reduction targets. Urban lifestyles are diverse and have a direct impact on energy consumption. The urbanization process can be viewed as a process of lifestyle choices. Promoting energy-efficient lifestyles through policy guidance in areas with high energy consumption can be beneficial as well. Appeals to use public transportation and green building, such as ultra-low-energy buildings and renewable energy technologies, are also important aspects of achieving low-carbon and sustainable development in the future [75,76,77].

### 4.3. Validity and Uncertainty of This Study

In this study, the impact analysis of UL on CEs at a single scale was abandoned, and we selected two high-precision grid scales of 5 km and 10 km for a comparative study. The differences in the impacts of UL on CEs at different scales were analyzed. The CE data in this study were obtained from the authoritative ODIAC database, which is a high-spatial-resolution global emission data product on CEs mainly based on fossil fuel combustion. Future studies can be conducted to calculate the carbon sources and sinks of the HREEB. Moreover, in this study, only a 5 km grid and a 10 km grid were selected to analyze the response mechanisms of UL on scale changes in CEs, and larger grid scales or administrative scales, such as those of village, town, and county, were not analyzed and verified. Therefore, it is necessary to further expand the range of scale selection in future research and explore appropriate evaluation scales for different research purposes. In addition, we can attempt to explain other aspects of the relationship between UL and CEs in the future, such as the coupling and coordination relationship.

## 5. Conclusions 

To reveal the impacts of UL on CEs in the HREEB from a multiscale perspective, this study first constructed a UL measurement method involving population, economy, and construction land. Then, based on a spatial autocorrelation analysis and GWR model, the spatial relationships and the impacts of UL on CEs from 2000 to 2020 were explored from a multiscale perspective. The results are summarized as follows:

(1) The total CEs in the HREEB witnessed an upsurge in the past two decades, with 1.664 × 10^7^ t in 2000 rocketing to 4.587 × 10^7^ t in 2020. Generally, CEs were mainly dispersed in the central urban areas of cities within the HREEB. Wuhan and Xiaogan in the east, Jingmen in the south, Xiangyang and Shiyan in the middle, Hanzhong in the west, and Nanyang in the north were the concentration areas of CEs.
(2) The ULs in different regions of the HREEB varied significantly, with high levels in the east and low levels in the central and western regions, while the overall UL in 2020 was higher than that in 2000, regardless of the research scale.
(3) During the study period, there was a significant, positive spatial correlation between UL and CEs, and similar spatial distribution characteristics of the bivariate spatial autocorrelation between CEs and UL at different times and different scales were observed.
(4) During the study period, UL had a positive correlation with CEs, but the impacts of UL on CEs varied at different grid scales. The regression coefficients in 2020 were higher than those in 2000, but the spatial distribution of the regression coefficients was more scattered, and more detailed information was provided at the 5 km grid scale than at the 10 km grid scale.

These findings are conducive to policy implications of low-carbon city development in areas with important ecological status, even for other similar ecological economic belts around the world.

## Data Availability

Not applicable.
